# Changes in Composition of Caecal Microbiota Associated with Increased Colon Inflammation in Interleukin-10 Gene-Deficient Mice Inoculated with *Enterococcus* Species

**DOI:** 10.3390/nu7031798

**Published:** 2015-03-11

**Authors:** Shalome A. Bassett, Wayne Young, Matthew P. G. Barnett, Adrian L. Cookson, Warren C. McNabb, Nicole C. Roy

**Affiliations:** 1 Food Nutrition & Health Team, Food & Bio-based Products Group, AgResearch Limited, Grasslands Research Centre, Tennent Drive, Palmerston North 4442, New Zealand; E-Mails: shalome.bassett@agresearch.co.nz (S.A.B.); wayne.young@agresearch.co.nz (W.Y.); nicole.roy@agresearch.co.nz (N.C.R.); 2 Nutrigenomics New Zealand, Private Bag 92019, Auckland 1142, New Zealand; E-Mail: warren.mcnabb@agresearch.co.nz; 3 Gravida: National Centre for Growth and Development, Private Bag 92019, Auckland 1142, New Zealand; 4 Food Assurance & Meat Quality Team, Food & Bio-based Products Group, AgResearch Grasslands, Tennent Drive, Palmerston North 4442, New Zealand; E-Mail: adrian.cookson@agresearch.co.nz; 5 Riddet Institute, Massey University, Tennent Drive, Palmerston North 4474, New Zealand; 6 AgResearch Grasslands, Tennent Drive, Palmerston North 4442, New Zealand

**Keywords:** Crohn’s disease, gastrointestinal disease, microbiome, colitis

## Abstract

Human inflammatory bowel disease (IBD) is a chronic intestinal disease where the resident microbiota contributes to disease development, yet the specific mechanisms remain unclear. Interleukin-10 gene-deficient (*Il10^-/-^*) mice develop inflammation similar to IBD, due in part to an inappropriate response to commensal bacteria. We have previously reported changes in intestinal morphology and colonic gene expression in *Il10^-/-^* mice in response to oral bacterial inoculation. In this study, we aimed to identify specific changes in the caecal microbiota associated with colonic inflammation in these mice. The microbiota was evaluated using pyrotag sequencing, denaturing gradient gel electrophoresis (DGGE) and quantitative real-time PCR. Microbiota profiles were influenced by genotype of the mice and by bacterial inoculation, and a strong correlation was observed between the microbiota and colonic inflammation scores. Although un-inoculated *Il10^-/-^* and C57 mice had similar microbiota communities, bacterial inoculation resulted in different changes to the microbiota in *Il10^-/-^* and C57 mice. Inoculated *Il10^-/-^* mice had significantly less total bacteria than un-inoculated *Il10^-/-^* mice, with a strong negative correlation between total bacterial numbers, relative abundance of *Escherichia/Shigella*, microbiota diversity, and colonic inflammation score. Our results show a putative causative role for the microbiota in the development of IBD, with potentially key roles for *Akkermansia*, or for *Bacteroides*, *Helicobacter*, *Parabacteroides*, and *Alistipes*, depending on the composition of the bacterial inoculum. These data support the use of bacterially-inoculated *Il10^-/-^* mice as an appropriate model to investigate human IBD.

## 1. Introduction

The term “inflammatory bowel disease” (IBD) refers to inappropriate and exaggerated immune-mediated disorders characterised by chronic and relapsing inflammation of the gastrointestinal tract, including Crohn’s disease (CD) and ulcerative colitis (UC). Studies suggest that dysbiosis, a harmful shift of the bacterial composition in the intestine, is a major factor in the pathogenesis of IBD, which, through interactions with genetic susceptibility factors, results in the dysregulation of the immune response to bacterial antigens [1,2,3,4,5,6,7]. It is clear that IBD is the consequence of inappropriate activation of the immune system and there is good evidence to show that the microbiota plays a role in the initiation, maintenance, and severity of IBD [8,9]. For example, inoculation with a simple consortium of seven human-derived IBD-related intestinal bacteria has been shown to induce colitis in germ-free interleukin-10 gene-deficient (*Il10^-/-^*) mice [8]. However, the specific contributions of the autochthonous microbial community have not yet been elucidated due to the complexity of such microbial ecosystems and a consistent microbial profile associated with IBD has yet to be identified [2,10].

Studies using animal models of intestinal inflammation such as the *Il10^-/-^* mouse have been crucial in extending our understanding of the pathogenesis of IBD, particularly to dissect the roles of specific bacteria and bacterial factors. For instance, *Il10^-/-^* mice do not develop colitis under germ-free conditions [11], develop inflammation of the colon only (colitis) when housed in specific-pathogen-free (SPF) environments [11,12], and develop chronic inflammation of the small intestine and colon (enterocolitis) when maintained in conventional conditions [12]. Intestinal inflammation in *Il10^-/-^* mice can also be induced by specific bacterial strains including enterococci [13,14,15], *E. coli* [14,15], or *Bacteroides vulgatus* [11]. These observations led us to examine whether inoculating *Il10^-/-^* mice (C57 background) with *Enterococcus* species would result in the development of intestinal inflammation, and therefore be an appropriate model of human IBD.

*Enterococcus* species are a common component of the intestinal microbiota of healthy humans and animals [11,16,17], with *E. faecalis* and *E. faecium* being the two species most commonly detected in the human bowel [18,19]. Both species are known to carry a variety of virulence factors (reviewed in [17]) which may play a role in the establishment of inflammation. In our previous studies, we observed no significant difference in colonic inflammation or colon gene expression levels between *Il10^-/-^* mice (C57 background) and C57 control mice maintained in either conventional or SPF conditions [20,21]. However, oral inoculation of these mice with intestinal bacteria, and in particular *Enterococcus* strains, increased colon inflammation compared with non-inoculated *Il10^-/-^* mice, and this inflammation was associated with changes in colon gene expression similar to those in human IBD, in particular CD [20].

In this study we tested the hypothesis that the increased colonic inflammation observed in *Il10^-/-^* mice receiving an oral bacterial inoculation corresponded with changes in the caecal microbiota of these animals. The caecum is an important repository for bacteria entering the colon, and changes in caecal bacterial profiles during the development of inflammation in this animal model have been shown to be the same as those occurring in both the proximal and distal colon in the same model [22]. We therefore used amplicon pyrotag sequencing of the 16S rRNA gene to compare the caecal bacterial composition from *Il10^-/-^* and C57 mice inoculated with a complex intestinal community (CIF), a mixture of *Enterococcus* strains (EF), or a combination of the two (EF.CIF), and un-inoculated mice housed in specific pathogen free (SPF) or conventional conditions (C). Specific changes in the caecal microbiota were then examined for correlations with previously reported colonic inflammation scores in these mice [20].

## 2. Experimental Section

### 2.1. Animal Experiments

All animal experiments were approved by the AgResearch Ruakura Animal Ethics Committee, Hamilton, New Zealand (application number 10339) operating under the New Zealand Animal Welfare Act 1999. The animals and experimental design used in this study have been previously described in detail [20]. Briefly, twenty five male *Il10^-/-^* mice (C57 background, Institute for Laboratory Animal Research designation B6.129P2-*Il10^tm1Cgn^*/J; Stock No. 002251) and twenty five male C57 control mice (Stock No. 000664) were received from The Jackson Laboratory (Bar Harbor, ME, USA) at approximately 5 weeks of age. *Il10^-/-^* mice were raised under maximum barrier (*i.e.*, essentially sterile) conditions prior to shipping, while C57 mice were raised under standard barrier conditions [23]. On arrival, mice were housed either in pairs or groups of three (5 mice per treatment) in shoebox-style cages. All mice were provided *ad libitum* access to water and AIN-76A powdered diet. The diet for all groups was sterilised by gamma irradiation (25 kGy, Schering-Plough, Wellington, New Zealand) to a level required for SPF conditions.

Both *Il10^-/-^* and C57 mice were randomly assigned to one of five treatment groups with five animals per group; one group of mice was housed in isolator cages (SPF); a second group was maintained under conventional conditions (C), and the remaining groups were kept in conventional conditions and orally inoculated with 12 *Enterococcus faecalis* and *E. faecium* strains listed in Table S1 (EF), conventional intestinal flora (CIF), or a 1:1 combination of the two (EF.CIF). Bacterial inoculations were given in a volume of 200 μL. CIF was derived from the intestinal contents of healthy age-matched C57BL/6 mice raised under conventional conditions, and was included to mimic the normal microbiota of healthy animals. Briefly, healthy, age-matched C57BL/6 mice were euthanised by CO_2_ asphyxiation and cervical dislocation, the gastrointestinal tract (from stomach to just below the caecum) removed, and digesta collected in sterile PBS, as previously described [20]. Each 200 μL of the EF inoculum contained 1.2 × 10^8^ colony forming units (CFU), while 200 μL of the EF.CIF inoculum included 6.0 × 10^7^ CFU from the EF inoculum. While the CIF inoculum has not been characterised (e.g., CFU within this inoculum were not assessed), the mice from which this was obtained were acquired from the same source for all subsequent studies using this protocol, and we have demonstrated a consistent effect on colon inflammation [20,24,25,26,27] and microbiota profile [28] in response to the EF.CIF inoculation. Bacterial solutions for oral inoculation (EF, CIF, and EF.CIF) were prepared as previously described [20].

### 2.2. Sample Collection

At 12 weeks of age, all mice were euthanised by CO_2_ asphyxiation and cervical dislocation. To minimise time variation between the last food intake and sampling, mice were fasted overnight (14 h) before sacrificing. On the morning of sampling, food was returned for 2 h followed by a further 2 h fast immediately prior to euthanasia upon which the caecum and its contents were removed from each mouse and frozen in liquid nitrogen.

### 2.3. Caecal Bacterial DNA Extraction

Metagenomic DNA was extracted from caecal contents using the QIAamp DNA stool minikit (Qiagen, Hilden, Germany) according to the manufacturer’s instructions.

### 2.4. Pyrotag Sequencing Analysis of Caecal Bacterial Composition

For pyrotag sequencing, the V4–V6 (V456) region of the bacterial 16S rRNA gene was amplified using forward primers containing the GS FLX A adapter sequence, an 8 nucleotide “barcode”, and template specific sequence (5′-CGTATCGCCTCCCTCGCGCCATCAG NNNNNNNN AGGCCAGCAGCCGCGGTAA-3′, with ‘N’ indicating barcode nucleotides), and reverse primers incorporating the GS FLX B adapter sequence and template specific sequence (5′-CTATGCGCCTTGCCAGCCCGCTCAG GCCRRCACGAGCTGACGAC-3′) [29]. The amplification mixture contained 50 μL of Roche FastStart PCR Master Mix (Roche, Basel, Switzerland), 2 μL of each primer (20 pmol/μL), 42 μL nuclease-free water, and 4 μL of cDNA or DNA template adjusted to a concentration of 50 ng/μL. Amplification was carried out on a MasterCycler ProS thermocycler (Eppendorf AG, Hamburg, Germany) using the following conditions 95 °C for 4 min, 25 cycles of (95 °C for 30 s; 49 °C for 30 s; 72 °C for 60 s) and 72 °C for 7 min. The expected PCR product size was 604 bp. Products were purified using Roche High Pure PCR Product Purification Kits according to the manufacturer’s instructions. Purified products were pooled in equal ng quantities and sent to Macrogen (Seoul, Korea) for unidirectional sequencing from the forward primer using the Roche GS-FLX sequencer with Titanium chemistry.

Sequences were quality filtered and de-multiplexed according to their barcode sequence using the Qiime 1.8 pipeline [30] with default settings. The resulting sequences were chimera checked using the USEARCH method against the Greengenes alignment (release GG_13_8), following which chimeric sequences were removed from subsequent analyses. Sequences showing 97% or greater similarity were clustered into operational taxonomic units (OTUs) using the UCLUST method and representative sequences were assigned taxonomies using the RDP classifier. Principal coordinate analysis (PCoA) of weighted UniFrac phylogenetic distances between communities, and Faith’s Phylogenetic Diversity estimates were performed using the core_diversity_analyses.py script in Qiime 1.8. Differences in mean proportions of taxa were analysed using the non-parametric Kruskal-Wallis test in R 3.0.2 [31], the results of which were corrected for multiple testing using the Benjamini and Hochberg false discovery rate (FDR) adjustment. FDR values <0.05 were considered significant. Significantly different groups were identified using the least significance difference (LSD) test in the agricolae package [32] for R. Spearman’s rho correlation (*r*) of bacterial taxa and previously published intestinal inflammation scores [20] were performed in R 3.0.2.

### 2.5. DGGE Analysis of Caecal Bacterial composition

The V2–V3 region of the 16S rRNA gene of bacteria in each sample was amplified using primers HDA1-GC (5ʹ-CGCCCGGGGCGCGCCCCGGGCGGGGCGGGGGCACGGGGGGACTCCTACGG GAGGCAGCAGT-3ʹ; underlined sequence indicates the GC clamp) and HDA-2 (5ʹ-GTATTACCG CGGCTGCTGGCA-3ʹ) as described by Tannock *et al*. [33]. PCR was performed as previously described [28] with the exception that PCR products were digested with Mung Bean nuclease prior to DGGE analysis according to the manufacturer’s instructions (Promega, Madison, WI, USA) to remove single stranded DNA fragments. DGGE separation of PCR products was performed using a Temporal Temperature Gel Electrophoresis mutation detection system (CBS Scientific Company Inc., Del Mar, CA, USA) in 6% polyacrylamide gels containing a 35 to 60% (w/v) gradient as described previously [34]. Electrophoresis was performed at constant voltage (65 V) and 60 °C for 14 h. Gels were stained with SYBR Gold (Invitrogen, CA, USA) and visualised by UV transillumination. DGGE profiles were compared from digital images of the gels using BioNumerics software (Applied Maths, Austin, TX, USA). Differences and similarities between the profiles were determining using the Dice similarity coefficient (*D_sc_*). A position tolerance of band matching of 1% was used for all analyses. Distances between samples were represented graphically by constructing a dendrogram using the Unweighted Pair Group Method with Arithmetic Mean (UPGMA) clustering. Principal component analysis (PCA) of band intensities was performed using R 3.0.2. Procrustes rotation analysis for comparing DGGE PCA scores to pyrotag sequence UniFrac distance PCoA scores were performed using the vegan package [35] in R. Procrustes analysis is a method for rotating and scaling points from one ordination (DGGE PCA scores) to be as close as possible to points from another ordination (UniFrac PCoA scores) while maintaining the relative distances between points within each ordination. The closer the ordination points between the different analyses are, the greater the correlation between the two datasets.

### 2.6. Real-Time PCR Quantification of Bacteria

The primers used for real-time PCR, the bacterial groups targeted, and the bacterial strains used for the preparation of the standard curves, are listed in Table S2. All bacterial strains except *E. coli* were grown anaerobically overnight at 37 °C. LB medium was used for growth of *E. coli* and de Man, Rogosa and Sharpe (MRS) broth (Oxoid, Hampshire, UK) supplemented with 0.05% (w/v) cysteine hydrochloride was used growth of *E. faecium*. Brain Heart Infusion broth (Oxoid) supplemented with 0.5% (w/v) yeast extract, 0.02% (w/v) cysteine hydrochloride, 10 mL per litre haemin (0.05% (w/v)) and 1 mL per litre Vitamin K solution (0.05% (v/v)) was used for the growth of *B. fragilis*. Bacterial cells were counted using a haemocytometer after 18 h incubation. Standard curves were prepared using genomic DNA extracted from a 10-fold dilution series (1 × 10^4^ to 1 × 10^9^ CFU/mL) of each bacterial reference strain as previously described [34]. Real-time quantitative PCR was carried out in triplicate on the Rotor-Gene™ 6000 (Corbett Research, Mortlake, NSW, Australia) using 15 μL reactions containing 7.5 μL of Express Sybr GreenER qPCR mix (Invitrogen), either 0.3 μM (for *E. faecium. B. fragilis*, and total bacteria (HDA-1/HDA-2)) or 0.45 μM (for *E. coli and B. vulgates*) of each primer (Table S2), and 75 ng of mouse caecal DNA template. Real-time PCR results were expressed as numbers of bacterial cells per g wet weight of caecal content and analysed by two factor analysis of variance (ANOVA) following log transformation in R 3.0.2, with *p* < 0.05 considered significant. Significant results were subsequently analysed using the LSD *post hoc* test.

## 3. Results

### 3.1. Inoculation Induced Dysbiosis of the Caecal Microbiota in Il10^-/-^ Mice

Pyrotag sequencing analysis of DNA extracted from caecal communities generated a total of 118,692 reads with a mean of 2525 reads per sample, a maximum of 4081, and a minimum of 887 reads, which was the value used for subsequent Faith’s Phylogenetic Diversity and UniFrac phylogenetic distance analyses.

**Figure 1:**
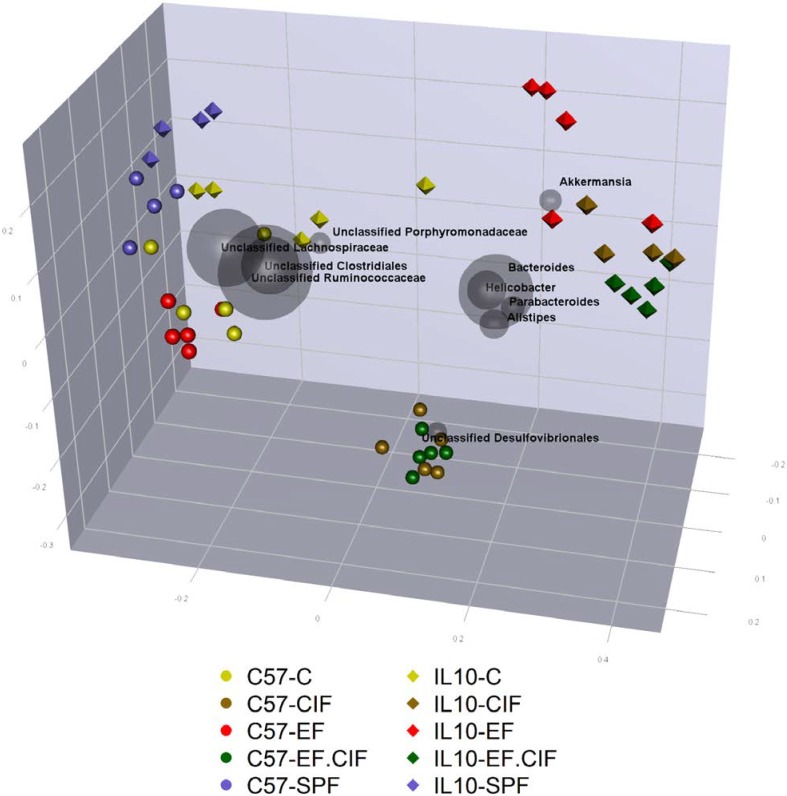
Principal coordinate analysis (PCoA) biplot of unweighted Unifrac phylogenetic distances between communities from C57 (spheres) and *Il10^-/-^* (tetrahedrons) mice inoculated with EF, CIF, or EF.CIF, or un-inoculated mice housed in specific pathogen free (SPF) or conventional (C) conditions. Distances between points indicate phylogenetic similarity of communities, with more similar communities placed closer together. Grey spheres indicate the ten most abundant taxa and their contribution to the separation between communities. The size of the grey spheres represent their relative abundance across all communities and the placement indicates which communities have the highest abundance of these taxa: the closer the community is to the taxa, the greater the relative proportions that taxa makes up of the total microbiota in that community.

**Figure 2:**
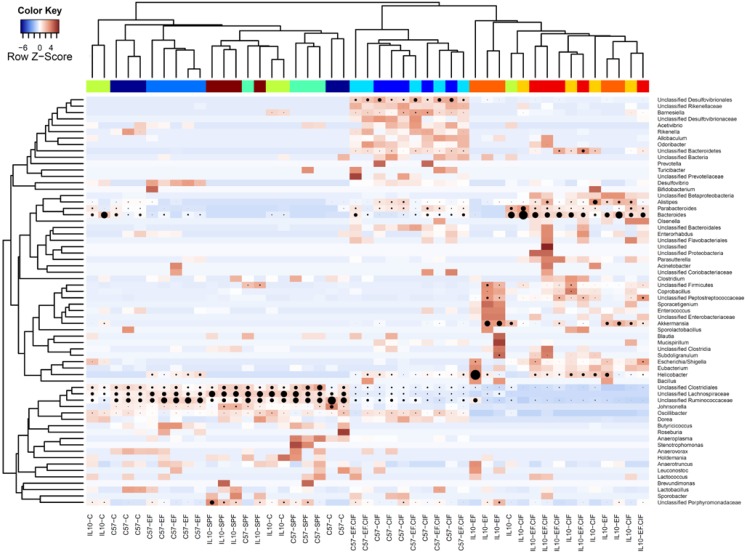
Heatmap showing hierarchical clustering of bacterial composition profiles from C57 and *Il10^-/-^* mice in treatment groups SPF, C, EF, CIF and EF.CIF. Taxa are indicated by row labels and samples are indicated by column labels. Heatmap colours (blue to dark red) signify relative prevalence of each taxon across samples and black circles show absolute proportions for each taxon within a sample, with circle size proportional to sequence abundance. The coloured bar beneath the upper dendrogram provides colour reference for identifying genotype and treatment group; all mice from the same group share the same colour.

UniFrac phylogenetic analysis showed that the caecal communities of C57 and *Il10^-/-^* mice under SPF conditions were highly similar (Figure 1). Likewise, the C57 and *Il10^-/-^* mice housed under convention conditions (C) showed only minor divergence (Figure 1 and Figure 2).

Despite the similarity of caecal communities between the two genotypes under SPF and conventional housing conditions, inoculation with bacteria resulted in very different responses from C57 and *Il10^-/-^* mice (Figure 1). Caecal communities of C57 mice inoculated with EF showed a small, but consistent change in the intestinal microbiota composition compared with un-inoculated, conventionally housed C57 mice (Figure 1 and Figure 2). In contrast, inoculating *Il10^-/-^* mice with EF led to a substantially altered caecal profile, which was driven by an increase in abundance of the genus *Akkermansia* (Figure 1). Whereas inoculation with EF had only a small effect on the caecal community of C57 mice, inoculation with CIF and EF.CIF resulted in a distinct shift in the microbiota of C57 mice, which was largely driven by an increased abundance of *Desulfovibrionales* (Figure 1). Inoculating *Il10^-/-^* mice with CIF or EF.CIF also led to a large shift in the caecal microbiota compared to un-inoculated *Il10^-/-^* mice. However, unlike with C57 mice, the shift in microbiota of *Il10^-/-^* mice driven by CIF and EF.CIF was mainly due to a relative increase in the genera *Bacteroides*, *Helicobacter*, *Parabacteroides*, and *Alistipes* (Figure 1 and Figure S1).

**Figure 3:**
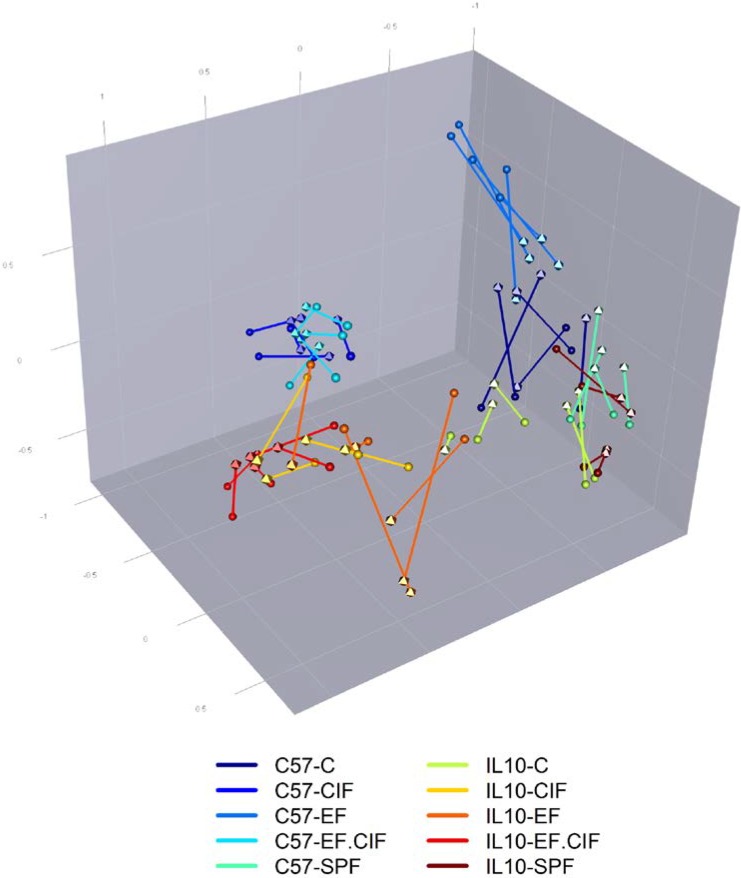
Procrustes rotation analysis comparing Unifrac phylogenetic distances from pyrotag sequencing and DGGE profiles of caecal communities of C57 and *Il10^-/-^* mice in treatment groups SPF, C, EF, CIF and EF-CIF. Procrustes analysis is a method for rotating and scaling points from one ordination to be as close as possible to points from another ordination, while maintaining the relative distances between points within each ordination. In this instance the ordination points are Unifrac PCoA scores (tetrahedrons) and DGGE profile PCA scores. Lines separating points indicates similarity of Unifrac and DGGE analysis of the same sample, with short lines indicating greater similarity. Lines and points are coloured according to genotype and treatment group.

The similarity of caecal microbiota profiles in mice inoculated with CIF and EF.CIF showed that the CIF inoculum had the most influential effect on the microbiota structure. The dominant effect of the CIF inoculum on the caecal community was supported by UPGMA clustering of caecal DGGE profiles (Figure S2), which also showed a clear delineation between mice that were inoculated with CIF or EF.CIF and those in different treatment groups. Procrustes rotation analysis showed that there was good concordance between UniFrac phylogenetic analysis of pyrotag sequences and DGGE profiles (*p* = 0.001; Figure 3).

**Figure 4:**
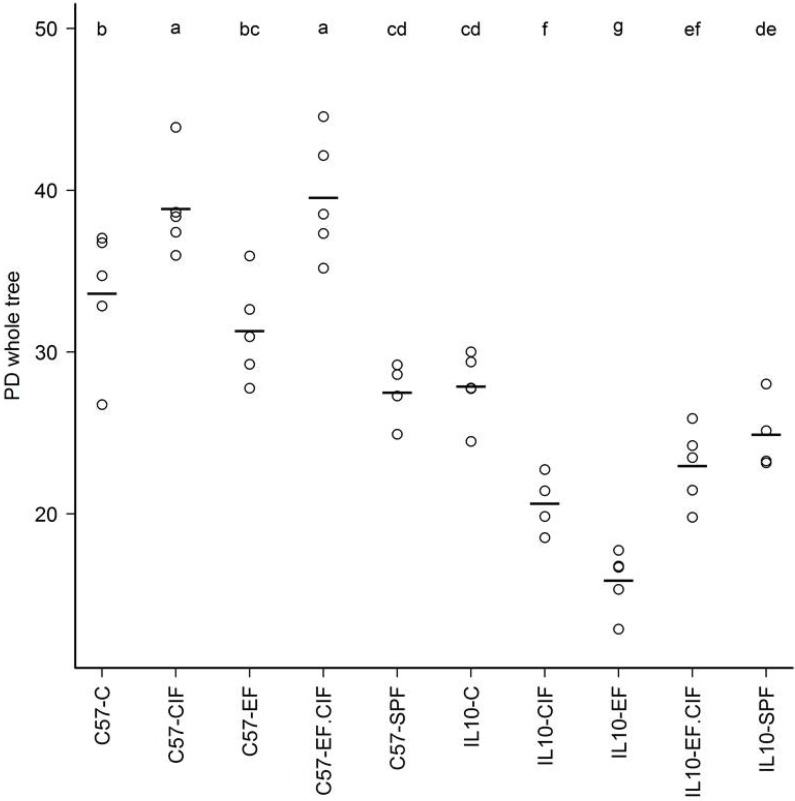
Faith’s Phylogenetic Diversity estimates of the caecal communities in C57 and *Il10^-/-^* mice in treatment groups SPF, C, EF, CIF and EF.CIF. The higher the estimate, the greater the diversity. Lines indicate mean estimates and groups that differ significantly (*p* < 0.05) are labelled with different letters. Mouse genotype/treatment groups are represented along the *x*-axis.

In addition to altering the proportions of bacterial groups present in the caecum, bacterial inoculation also changed the diversity of the community (Figure 4). However, these effects varied depending on mouse genotype. Bacterial inoculation with CIF and EF.CIF increased caecal diversity in C57 mice, whereas *Il10^-/-^* mice inoculated with CIF, EF, and EF.CIF showed decreased caecal diversity compared to un-inoculated conventionally housed *Il10^-/-^* mice (Figure 4).

**Figure 5:**
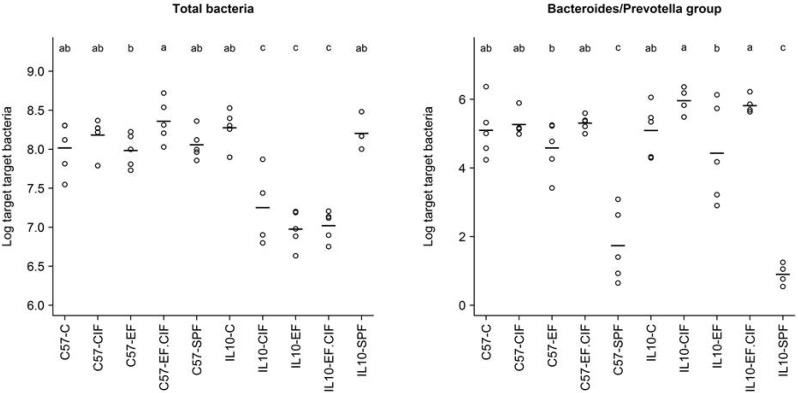

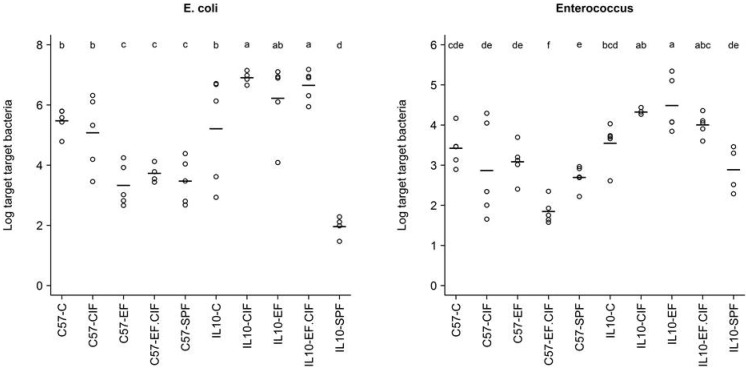
qPCR quantification of the total number of bacterial cells in the caecum of *Il10^-/-^* and C57 mice housed in either specific pathogen free (SPF) conditions or maintained under conventional conditions (C) and also receiving either an oral bacterial inoculation of 12 *E. faecalis* and *E. faecium* strains (EF), complex intestinal flora collected from healthy control mice (CIF), or a 50:50 mixture of the two (EF.CIF). The horizontal lines represent the mean bacterial cell numbers of the individual mice; cell numbers of bacteria that differ significantly between groups (*p* < 0.05), when comparing between mice from the same genotype in different treatment groups are labelled with different letters. Mouse genotype/treatment groups are represented along the *x*-axis.

The bacterial inoculation-induced dysbiosis detected by pyrotag sequencing and DGGE were followed up by qPCR quantification of total bacteria, *Bacteroides/Prevotella*, *E. coli*, and *Enterococcus* abundance (Table 1). Bacterial inoculation had no effect on the total number of caecal bacteria in C57 mice (Figure 5). However, inoculating *Il10^-/-^* mice with EF, EF.CIF or CIF led to a significant decline (*p* < 0.001) in the total number of caecal bacteria (Figure 5). On the whole, inoculation with EF and EF.CIF did not increase the caecal abundance of *Enterococcus*, as detected by qPCR. However, a notable exception was in *Il10^-/-^* mice, where EF inoculation led to significant increase (*p* < 0.001) in *Enterococcus* numbers compared to un-inoculated conventionally housed *Il10^-/-^* mice (Figure 5). The correlation between the abundance of *Enterococcus* detected by qPCR and *Enterococcus* proportions detected by pyrotag sequencing was remarkably strong, with a Spearman Rho coefficient of 0.81. Similarly, there were high correlations between qPCR abundance of *E. coli* and sequencing *Escherichia/Shigella* proportions (Spearman Rho = 0.88), and between qPCR abundance of *Bacteroides/Prevotella* group and sequencing *Bacteroides* proportions (Spearman Rho = 0.94).

**Table 1:** Statistical analysis of bacterial group qPCR results. Significance values of bacterial qPCR results examining the effect of genotype, treatment, and the interaction between genotype and treatment on the number of bacteria present in the caecum (2 factor ANOVA). *Bacteroides/Prevotella*, *E. coli*, and *Enterococcus* were selected because of evidence for changes in these groups from either sequencing or DGGE analysis. For each target bacterial group, analysis was performed on log_10_ target bacteria numbers per 10^7^ total bacteria. For total bacteria, analysis was performed on log_10_ total bacteria numbers per 75 ng DNA. *p* < 0.05 was considered statistically significant.

	*Bacteroides*-*Prevotella* Group	*E. coli*	*Enterococcus*	Total Bacteria
Genotype	0.56	6.33E-05	1.24E-07	3.59E-09
Treatment	1.80E-14	2.75E-07	2.70E-03	5.95E-06
Genotype: Treatment	0.25	1.13E-05	1.25E-03	2.69E-08

### 3.2. Inflammation-Associated Bacteria

**Figure 6:**
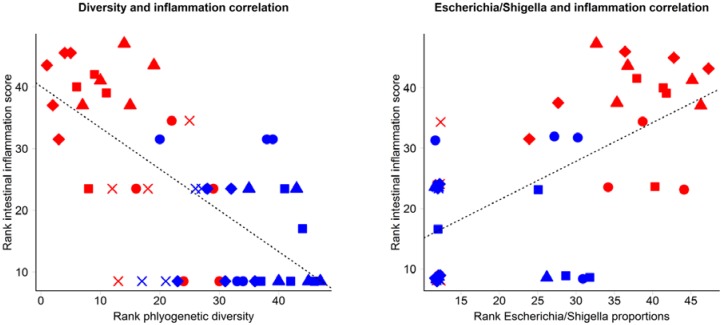

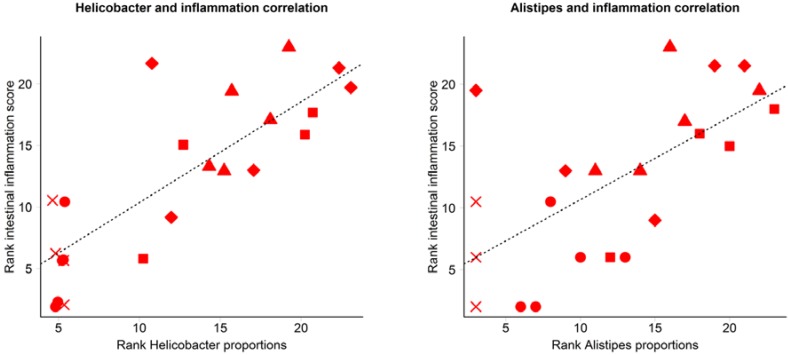
Correlation between rank of intestinal inflammation scores reported previously [20,21] and rank of proportions of selected bacterial taxa identified from pyrotag sequencing across all inoculation treatments in C57 and *Il10^-/-^* mice. Dotted line indicates linear regression. Blue points indicate C57 mice and red points indicate *Il10^-/-^* mice. Shapes of the points indicate treatment groups: SPF (×), C (circle), CIF (square), EF (diamond), and EF.CIF (triangle).

Several aspects of the bacterial inoculation induced dysbiosis seen in *Il10^-/-^* mice were associated with the severity of inflammation, which we have previously reported [21]. A strong negative correlation was observed between caecal diversity and inflammation score (*r* = −0.65; Figure 6). Abundance of *Escherichia/Shigella* from pyrotag sequence analysis and inflammation scores showed a strong positive correlation (*r* = 0.66). A strong association was also seen between *Helicobacter* proportions and inflammation (*r* = 0.58), which was very strong when the observations were restricted to *Il10^-/-^* mice only (*r* = 0.84; Figure 6). Among *Il10^-/-^* mice, prevalence of *Alistipes* was also highly correlated with intestinal inflammation (*r* = 0.67; Figure 6). However, despite the prevalence of intestinal inflammation seen in *Il10^-/-^* mice inoculated with EF and EF.CIF, there was only a moderate correlation between *Enterococcus* proportions and inflammation scores (*r* = 0.44).

## 4. Discussion

Our results show that an oral inoculation with a bacterial preparation (EF, CIF or EF.CIF) caused major changes in the caecal microbiota of *Il10^-/-^* mice compared with un-inoculated (C and SPF) *Il10^-/-^* mice. Bacterial inoculation also affected the microbiota of C57 mice, but in a different way compared to *Il10^-/-^* mice. Specific changes observed in the microbiota were associated with severity of inflammation in *Il10^-/-^* mice, providing support for the role of dysbiosis in the pathogenesis of IBD.

In our study we examined the effects of oral inoculation with bacteria known to have inflammatory effects (EF), a complex community of large bowel derived bacteria from healthy donors (CIF), or a mixture of both (EF.CIF). We have previously reported that all three inocula caused inflammation in *Il10^-/-^* mice, but not in C57 mice [20,21]. Although inoculation with *E. faecalis* has been shown to induce inflammation in germ-free *Il10^-/-^* mice [14,15], we have demonstrated that inoculation with *Enterococcus* (EF) can also induce inflammation in *Il10^-/-^* mice with a resident microbiota. Furthermore, we have shown that this resident microbiota is very similar to that of wild type C57 mice at 5 weeks of age. While it is perhaps not surprising that inoculation with potential pathogens can cause inflammation, it is clear that inoculation with the intestinal community from healthy donors (CIF) can also cause inflammation. Considering the interest in faecal transplantation as a therapy for inflammatory conditions such as *Clostridium difficile* infection [36], our results suggest care is required when selecting suitable donors. Furthermore, we show that a single inoculation of CIF can lead to an altered microbiota in healthy wild-type C57 mice which is still apparent even after 7 weeks, which raises the possibility of relatively long-term changes to the microbiota after just a single event.

It is clear from our study that the introduction of exogenous bacteria led to induction of sustained intestinal inflammation. Whether the inflammation was caused by the bacteria from the inoculum or from changes in the microbial ecosystem following the introduction of inoculum-derived bacteria remains to be determined. It has been proposed that dysbiosis is secondary to the immune response in the *Il10^-/-^* model [37]. This suggests that in our study, inoculation with exogenous bacteria triggers the immune response, causing inflammation which is followed by dysbiosis. The altered microbiota profiles may then act to perpetuate and amplify the inflammatory response [37]. It is also possible that both the initial perturbation and consequent dysbiosis play a role in the pathogenesis of intestinal inflammation. We have previously reported that inoculation of *Il10^-/-^* mice with *Enterococcus* only (EF) resulted in severe intestinal inflammation that was of similar severity to *Il10^-/-^* mice that had received CIF or EF.CIF [21]. However, the prevalence of *Enterococcus* in the caecum at the end of the study period did not correlate well with the inflammation scores, suggesting that the combined activity of other members of the community contribute to the severity of inflammation. On the other hand, taxonomic groups that did correlate well with inflammation scores in our study have also been shown in other studies to cause, exacerbate, or are otherwise implicated in intestinal inflammation, such as *Escherichia coli*, *Alistipes*, and *Helicobacter* [8,37,38,39,40].

Considering the impact that the microbiota has on inflammation, it may seem paradoxical that the increased severity of inflammation was associated with a decrease in caecal diversity and absolute numbers of bacteria. However, this reduction in diversity associated with intestinal inflammation has been observed in a number of other studies [41,42]. Although the reduction in absolute numbers of caecal bacteria has been less well reported, a reduction in numbers of faecal commensal bacteria in UC and CD patients has also been described [43]. Together, these observations suggest that reduced biodiversity and total numbers of the resident microbiota may expose a genetically susceptible individual to a greater risk of inflammation as the resident intestinal community is less able to recover from disruptions to homeostasis. This could also result in physical exposure of the intestinal cells to either pathogens or environmental triggers. Either or both of these factors may in turn may lead to a disproportionate influence of potentially harmful bacteria such as *Helicobacter* and *E. coli*, either due to a change in balance in the competition for resources or the new availability of previously occupied ecological niches. Indeed, the removal of commensal bacteria through the use of antibiotics has been shown to facilitate the expansion of *C. difficile* and *Salmonella enterica* serovar Typhimurium by removing competition for host-derived sugars such as sialic acid [44]. It therefore seems prudent to consider the importance of the collective microbiota as a whole, rather than only focussing on any particular taxa, especially in light of our results showing that the effects of a complex community (CIF) appear to dominate the effects of a less diverse inoculum (EF), depending on the genetic background of the host.

## 5. Conclusions

In summary, we have described changes in the microbiota associated with intestinal inflammation in a mouse model of human IBD. The results of our study demonstrate that the oral inoculation of *Il10^-/-^* mice with intestinal bacteria results in substantial changes in the caecal microbiota both in quantitative as well as in qualitative terms, and that these changes correlate with the severity of inflammation.

## Supplementary Files

Supplementary File 1

## References

[1] Conte M.P., Schippa S., Zamboni I., Penta M., Chiarini F., Seganti L., Osborn J., Falconieri P., Borrelli O., Cucchiara S. (2006). Gut-associated bacterial microbiota in paediatric patients with inflammatory bowel disease. Gut.

[2] Frank D.N., St. Amand A.L., Feldman R.A., Boedeker E.C., Harpaz N., Pace N.R. (2007). Molecular-phylogenetic characterization of microbial community imbalances in human inflammatory bowel diseases. Proc. Natl. Acad. Sci. USA.

[3] Gueimonde M., Ouwehand A., Huhtinen H., Salminen E., Salminen S. (2007). Qualitative and quantitative analyses of the bifidobacterial microbiota in the colonic mucosa of patients with colorectal cancer, diverticulitis and inflammatory bowel disease. World J. Gastroenterol..

[4] Lepage P., Hösler R., Spehlmann M.E., Rehman A., Zvirbliene A., Begun A., Ott S., Kupcinskas L., Doré J., Raedler A. (2011). Twin study indicates loss of interaction between microbiota and mucosa of patients with ulcerative colitis. Gastroenterology.

[5] Noor S.O., Ridgway K., Scovell L., Kemsley E.K., Lund E.K., Jamieson C., Johnson I.T., Narbad A. (2010). Ulcerative colitis and irritable bowel patients exhibit distinct abnormalities of the gut microbiota. BMC Gastroenterol..

[6] Ott S.J., Musfeldt M., Wenderoth D.F., Hampe J., Brant O., Fölsch U.R., Timmis K.N., Schreiber S. (2004). Reduction in diversity of the colonic mucosa associated bacterial microflora in patients with active inflammatory bowel disease. Gut.

[7] Takaishi H., Matsuki T., Nakazawa A., Takada T., Kado S., Asahara T., Kamada N., Sakuraba A., Yajima T., Higuchi H. (2008). Imbalance in intestinal microflora constitution could be involved in the pathogenesis of inflammatory bowel disease. Int. J. Med. Microbiol..

[8] Eun C.S., Mishima Y., Wohlgemuth S., Liu B., Bower M., Carroll I.M., Sartor R.B. (2014). Induction of bacterial antigen-specific colitis by a simplified human microbiota consortium in gnotobiotic interleukin-10^-/-^ mice. Infect. Immun..

[9] Sartor R.B., Mazmanian S.K. (2012). Intestinal Microbes in Inflammatory Bowel Diseases. Am. J. Gastroenterol. Suppl..

[10] Frank D., Robertson C., Hamm C., Kpadeh Z., Zhang T., Chen H., Zhu W., Sartor R., Boedeker E., Harpaz N. (2011). Disease phenotype and genotype are associated with shifts in intestinal-associated microbiota in inflammatory bowel diseases. Inflamm. Bowel Dis..

[11] Sellon R.K., Tonkonogy S., Schultz M., Dieleman L.A., Grenther W., Balish E., Rennick D.M., Sartor R.B. (1998). Resident enteric bacteria are necessary for development of spontaneous colitis and immune system activation in interleukin-10-deficient mice. Infect. Immun..

[12] Kuhn R., Lohler J., Rennick D., Rajewsky K., Muller W. (1993). Interleukin-10-deficient mice develop chronic enterocolitis. Cell.

[13] Balish E., Warner T. (2002). *Enterococcus faecalis* induces inflammatory bowel disease in interleukin-10 knockout mice. Am. J. Pathol..

[14] Kim S.C., Tonkonogy S.L., Albright C.A., Tsang J., Balish E.J., Braun J., Huycke M.M., Sartor R.B. (2005). Variable phenotypes of enterocolitis in interleukin 10-deficient mice monoassociated with two different commensal bacteria. Gastroenterology.

[15] Kim S.C., Tonkonogy S.L., Karrasch T., Jobin C., Sartor R.B. (2007). Dual-association of gnotobiotic *IL-10^-/-^* mice with 2 nonpathogenic commensal bacteria induces aggressive pancolitis. Inflamm. Bowel Dis..

[16] Eaton T.J., Gasson M.J. (2001). Molecular screening of *Enterococcus* virulence determinants and potential for genetic exchange between food and medical isolates. Appl. Environ. Microbiol..

[17] Jett B.D., Huycke M.M., Gilmore M.S. (1994). Virulence of enterococci. Clin. Microbiol. Rev..

[18] Finegold S.M., Sutter V.L., Mathisen G.E., Hentges D.J. (1983). Normal indigenous intestinal flora. Human Intestinal Microflora in Health and Disease.

[19] Tannock G.W., Cook G., Gilmore M.S., Clewell D.B., Courvalin P.M., Dunny G.M., Murray B.E., Rice L.B. (2002). Enterococci as members of the intestinal microflora of humans. The Enterococci: Pathogenesis, Molecular Biology, and Antibiotic Resistance.

[20] Barnett M.P., McNabb W.C., Cookson A.L., Zhu S., Davy M., Knoch B., Nones K., Hodgkinson A.J., Roy N.C. (2010). Changes in colon gene expression associated with increased colon inflammation in interleukin-10 gene-deficient mice inoculated with *Enterococcus* species. BMC Immunol..

[21] Roy N.C., Barnett M.P.G., Knoch B., Dommels Y.E.M., McNabb W.C. (2007). Nutrigenomics applied to an animal model of Inflammatory Bowel Diseases: Transcriptomic analysis of the effects of eicosapentaenoic acid- and arachidonic acid-enriched diets. Mutat. Res..

[22] Bibiloni R., Simon M., Albright C.A., Sartor B., Tannock G. (2005). Analysis of the large bowel microbiota of colitic mice using PCR/DGGE. Lett. Appl. Microbiol..

[23] Laboratory T.J. Overview of the Jackson Laboratory Facility Barrier Levels. http://jaxmice.jax.org/health/barrier.html.

[24] Knoch B., Barnett M.P., Cooney J., McNabb W.C., Barraclough D., Laing W., Zhu S., Park Z.A., Maclean P., Knowles S.O. (2010). Molecular characterization of the onset and progression of colitis in inoculated interleukin-10 gene-deficient mice: A role for PPARalpha. PPAR Res..

[25] Knoch B., Barnett M.P., McNabb W.C., Zhu S., Park Z.A., Khan A., Roy N.C. (2010). Dietary arachidonic acid-mediated effects on colon inflammation using transcriptome analysis. Mol. Nutr. Food. Res..

[26] Knoch B., Barnett M.P.G., Zhu S.T., Park Z.A., Nones K., Dommels Y.E.M., Knowles S.O., McNabb W.C., Roy N.C. (2009). Genome-wide analysis of dietary eicosapentaenoic acid- and oleic acid-induced modulation of colon inflammation in interleukin-10 gene-deficient mice. J. Nutrigenet. Nutrigenomics.

[27] Russ A.E., Peters J.S., McNabb W.C., Barnett M.P.G., Anderson R.C., Park Z., Zhu S.T., Maclean P., Reynolds G.W., Roy N.C. (2013). Gene expression changes in the colon epithelium are similar to those of intact colon in the interleukin-10 gene deficient mouse in late inflammation. PloS One.

[28] Knoch B., Nones K., Barnett M.P., McNabb W.C., Roy N.C. (2010). Diversity of caecal bacteria is altered in interleukin-10 gene-deficient mice before and after colitis onset and when fed polyunsaturated fatty acids. Microbiology.

[29] Claus S.P., Ellero S.L., Berger B., Krause L., Bruttin A., Molina J., Paris A., Want E.J., de Waziers I., Cloarec O. (2011). Colonization-induced host-gut microbial metabolic interaction. MBio.

[30] Caporaso J.G., Kuczynski J., Stombaugh J., Bittinger K., Bushman F.D., Costello E.K., Fierer N., Pena A.G., Goodrich J.K., Gordon J.I. (2010). QIIME allows analysis of high-throughput community sequencing data. Nat. Methods.

[31] R Core Team (2010). R: A language and Environment for Statistical Computing.

[32] De Mendiburu F. Agricolae: Statistical Procedures for Agricultural Research. http://CRAN.R-project.org/package=agricolae.

[33] Tannock G.W., Munro K., Harmsen H.J., Welling G.W., Smart J., Gopal P.K. (2000). Analysis of the fecal microflora of human subjects consuming a probiotic product containing *Lactobacillus rhamnosus* DR20. Appl. Environ. Microbiol..

[34] Nones K., Knoch B., Dommels Y.E., Paturi G., Butts C., McNabb W.C., Roy N.C. (2009). Multidrug resistance gene deficient (*mdr1a*^-/-^) mice have an altered caecal microbiota that precedes the onset of intestinal inflammation. J. Appl. Microbiol..

[35] Oksanen J., Blanchet F.G., Kindt R., Legendre P., Minchin P.R., O’Hara R.B., Simpson G.L., Solymos P., Stevens M.H.H., Wagner H. Vegan: Community Ecology Package. http://CRAN.R-project.org/package=vegan.

[36] Bakken J.S., Borody T., Brandt L.J., Brill J.V., Demarco D.C., Franzos M.A., Kelly C., Khoruts A., Louie T., Martinelli L.P. (2011). Treating *Clostridium difficile* infection with fecal microbiota transplantation. Clin. Gastroenterol. Hepatol..

[37] Maharshak N., Packey C.D., Ellermann M., Manick S., Siddle J.P., Huh E.Y., Plevy S., Sartor R.B., Carroll I.M. (2013). Altered enteric microbiota ecology in interleukin 10-deficient mice during development and progression of intestinal inflammation. Gut Microbes.

[38] Winter S.E., Winter M.G., Xavier M.N., Thiennimitr P., Poon V., Keestra A.M., Laughlin R.C., Gomez G., Wu J., Lawhon S.D. (2013). Host-derived nitrate boosts growth of *E. coli* in the inflamed gut. Science.

[39] Morgan X.C., Tickle T.L., Sokol H., Gevers D., Devaney K.L., Ward D.V., Reyes J.A., Shah S.A., LeLeiko N., Snapper S.B. (2012). Dysfunction of the intestinal microbiome in inflammatory bowel disease and treatment. Genome Biol..

[40] Yang I., Eibach D., Kops F., Brenneke B., Woltemate S., Schulze J., Bleich A., Gruber A.D., Muthupalani S., Fox J.G., Josenhans C. (2013). Intestinal microbiota composition of interleukin-10 deficient C57BL/6J mice and susceptibility to *Helicobacter hepaticus*-induced colitis. PLoS One.

[41] Manichanh C., Rigottier-Gois L., Bonnaud E., Gloux K., Pelletier E., Frangeul L., Nalin R., Jarrin C., Chardon P., Marteau P. (2006). Reduced diversity of faecal microbiota in Crohn's disease revealed by a metagenomic approach. Gut.

[42] Lapthorne S., Pereira-Fantini P.M., Fouhy F., Wilson G., Thomas S.L., Dellios N.L., Scurr M., O'Sullivan O., Ross R.P., Stanton C. (2013). Gut microbial diversity is reduced and is associated with colonic inflammation in a piglet model of short bowel syndrome. Gut Microbes.

[43] Swidsinski A., Loening-Baucke V., Vaneechoutte M., Doerffel Y. (2008). Active Crohn's disease and ulcerative colitis can be specifically diagnosed and monitored based on the biostructure of the fecal flora. Inflamm. Bowel Dis..

[44] Ng K.M., Ferreyra J.A., Higginbottom S.K., Lynch J.B., Kashyap P.C., Gopinath S., Naidu N., Choudhury B., Weimer B.C., Monack D.M. (2013). Microbiota-liberated host sugars facilitate post-antibiotic expansion of enteric pathogens. Nature.

