# Supplementary material for: Changes in Composition of Caecal Microbiota Associated with Increased Colon Inflammation in Interleukin-10 Gene-Deficient Mice Inoculated with Enterococcus Species
Source: Nutrients. 2015 Mar 11;7(3):1798–816. doi: 10.3390/nu7031798 (PMC4377882; doi:10.3390/nu7031798)
Supplement: Supplementary File 1 [file nutrients-07-01798-s001.docx]

Supplementary Information

**Table S1.** Strains of *Enterococcus faecalis* and *E. faecium* used in solutions for oral inoculation with EF and EF.CIF.

| ***Enterococcus faecalis*** | | ***Enterococcus faecium*** | |
| --- | --- | --- | --- |
| **Strain** | **Source** | **Strain** | **Source** |
| AGR991 | calf | SN081 | calf |
| AGR1140 | calf | SN068 | calf |
| AGR1371 | calf | SN077 | calf |
| SN070 | poultry | SN067 | calf |
| SN079 | poultry | SN071 | calf |
| SN083 | poultry | AGR979 | poultry |

Reproduced with permission from Barnett *et al*., (2010) [1].

**Table S2.** Strains and oligonucleotide sequences used for quantification of selected bacterial groups by qPCR.

| **Target Organism** | **Strain Used for Standard Curve** | **Primers** | **Sequence 5ʹ–3ʹ** | **Reference** |
| --- | --- | --- | --- | --- |
| Total bacteria | *E. coli* AGR681 | HDA-1  HDA-2 | ACTCCTACGGGAGGCAGCAG GTATTACCGCGGCTGCTGGCA | [2] |
| *Clostridium  leptum group* | *C. leptum* DSM753 | F_Clept  R_Clept | CCTTCCGTGCCGSAGTTA  GAATTAAACCACATACTC CACTGCTT | [3] |
| *Bifidobacterium* | *B. animalis subsp. lactis Bb12* | F_Bifid  R_Bifid | CGGGTGAGTAATGCGTGACC  TGATAGGACGCGACCCCA | [3] |
| *E. coli* | *E. coli* AGR681 | *E. coli* F  *E. coli* R | CATGCCGCGTGTATGAAGAA  CGGGTAACGTCAATGAGCAAA | [4] |
| *Enterococcus* | *E. faecium* ATCC19434 | F_Entero  R_Entero | CCCTTATTGTTAGTTGCCATCATT  ACTCGTTGTACTTCCCATTGT | [5] |
| *Bacteroides/*  *Prevotella group* | *B. fragilis* DSM2151 | F_Bacter 11  R_Bacter 08 | CCTWCGATGGATAGGGGTT  CACGCTACTTGGCTGGTTCAG | [3] |
| *Lactobacillus/*  *Leuconostoc/*  *Pediococcus group* | *L. reuteri* 20016 | F_Lacto 05  R_Lacto 04 | AGCAGTAGGGAATCTTCCA  CGCCACTGGTGTTCYTCCATATA | [3] |


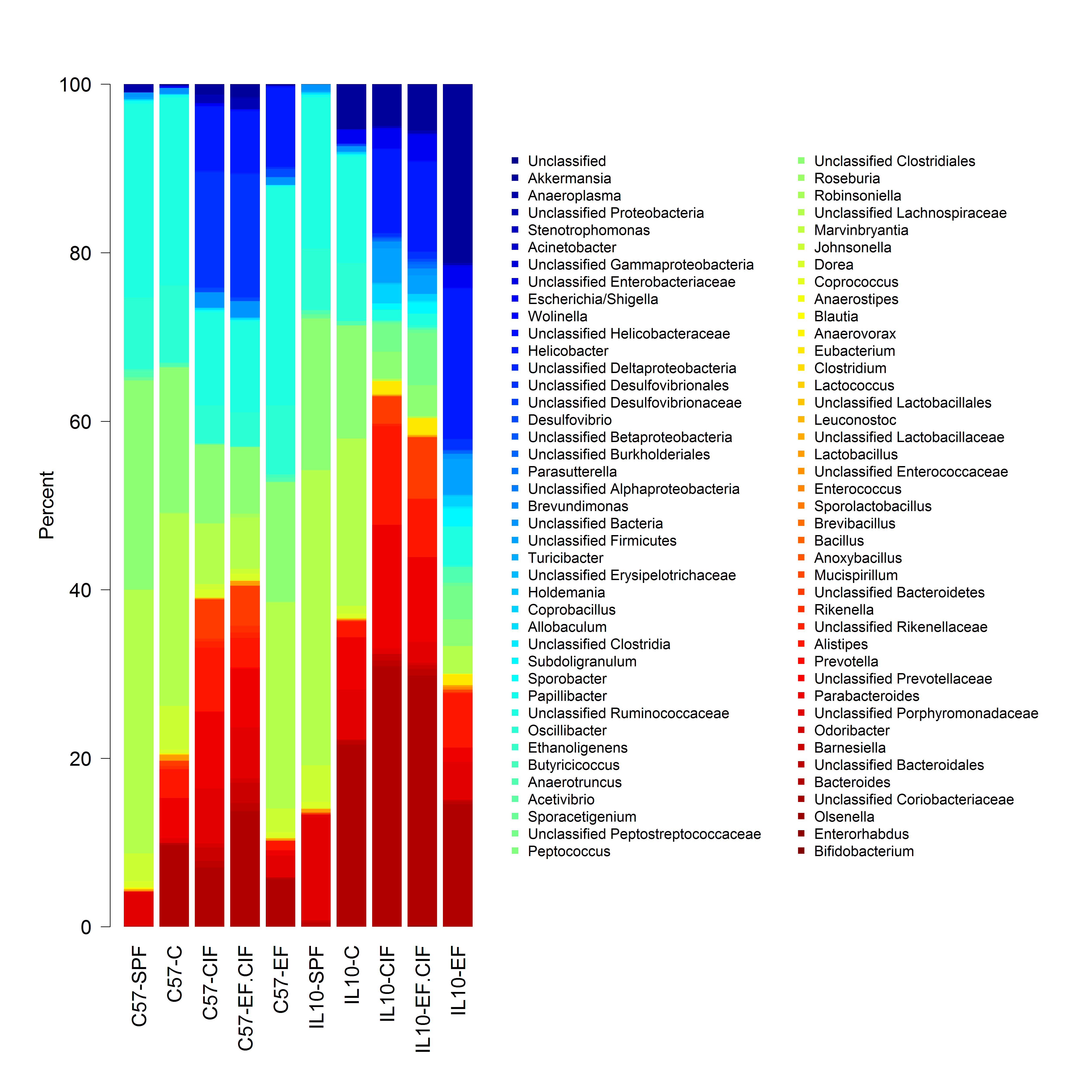


**Figure S1.** Stacked barplot showing mean proportions of genus level taxa in *Il10*^-/-^ and C57 mice. Mice of each genotype were randomly assigned to one of five treatment groups (*n* = 5 per group); housed in specific pathogen free (SPF) conditions, maintained under conventional conditions (C), or maintained under conventional conditions and receiving a single oral bacterial inoculation of either 12 *Enterococcus faecalis* and *E. faecium* strains (EF), complex intestinal flora collected from healthy control mice (CIF), or a 50:50 mixture of the two (EF.CIF).


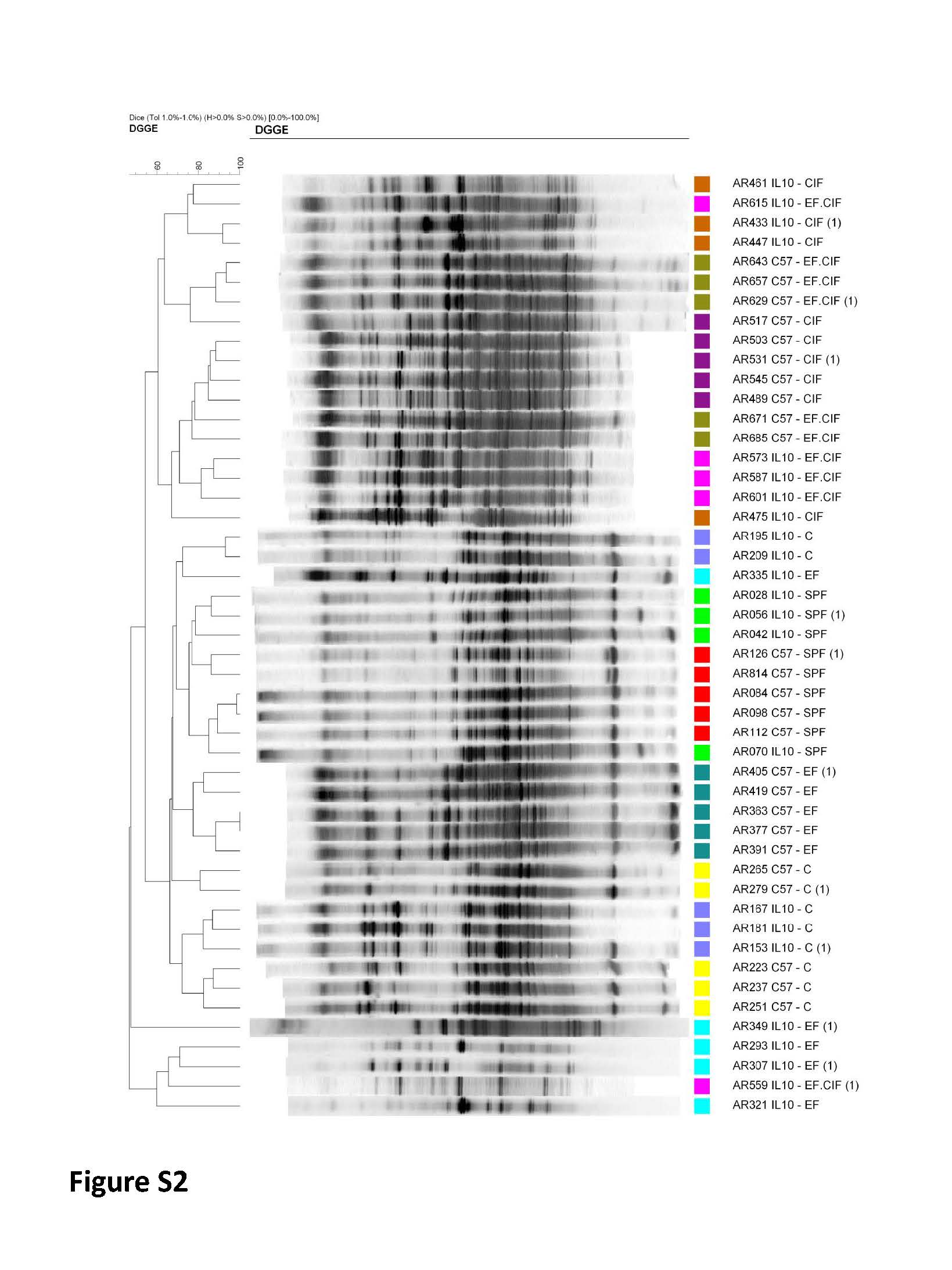


**Figure S2.** DGGE profiles of caecal microbiota from individual *Il10*^-/-^ and C57 mice. Mice were housed in specific pathogen free (SPF) conditions, maintained under conventional conditions (C), or maintained under conventional conditions and also received a single oral bacterial inoculation of either 12 *Enterococcus faecalis* and *E. faecium* strains (EF), complex intestinal flora collected from healthy control mice (CIF), or a 50:50 mixture of the two (EF.CIF). Dice’s similarity coefficient (*D_SC_*) using a band tolerance of 1% and a dendogram depicting the clustering analysis is also shown. The scale bar of the dendogram indicates the % similarity (*D_SC_*) between the samples with respect to their DGGE profile.

References

1. Barnett, M.P.; McNabb, W.C.; Cookson, A.L.; Zhu, S.; Davy, M.; Knoch, B.; Nones, K.; Hodgkinson, A.J.; Roy, N.C. Changes in colon gene expression associated with increased colon inflammation in interleukin-10 gene-deficient mice inoculated with *Enterococcus* species. *BMC Immunol.* **2010**, *11*, doi:10.1186/1471-2172-11-39.
2. Tannock, G.W.; Munro, K.; Harmsen, H.J.; Welling, G.W.; Smart, J.; Gopal, P.K. Analysis of the fecal microflora of human subjects consuming a probiotic product containing *Lactobacillus rhamnosus* DR20. *Appl. Environ. Microbiol.* **2000**, *66*, 2578–2588.
3. Furet, J.P.; Firmesse, O.; Gourmelon, M.; Bridonneau, C.; Tap, J.; Mondot, S.; Doré, J.;
   Corthier, G. Comparative assessment of human and farm animal faecal microbiota using real-time quantitative PCR. *FEMS Microbiol. Ecol.* **2009**, *68*, 351–362.
4. Huijsdens, X.W.; Linkens, R.K.; Mak, M.; Neuwissen, S.G.; Vanderbroucke-Grauls, C.M.; Savelkoul, P.H. Quantification of bacteria adherent to gastrointestinal mucosa by real-time PCR.
   *J. Clin. Microbiol.* **2002**, *40*, 4423–4427.
5. Rinttilä, T.; Kassinen, A.; Malinen, E.; Kroguis, L.; Palva, A. Development of an extensive set of 16S rDNA-targeted primers for quantification of pathogenic and indigenous bacteria in faecal samples by real-time PCR. *J. Appl. Microbiol.* **2004**, *97*, 1166–1177.

© 2015 by the authors; licensee MDPI, Basel, Switzerland. This article is an open access article distributed under the terms and conditions of the Creative Commons Attribution license (http://creativecommons.org/licenses/by/4.0/).
